# The Happy Child Program’s Intersectionality: Prenatal Home Visit Frequency, Food Insecurity Risk, Symptoms of Depression, and Parental Practices in Brazilian Women Assisted during Pregnancy

**DOI:** 10.3390/nu16172990

**Published:** 2024-09-04

**Authors:** Camila Biete, Vivian S. S. Gonçalves, Ariene S. Carmo, Nathalia Pizato

**Affiliations:** 1 Graduate Program in Human Nutrition, Department of Nutrition, University of Brasilia, Brasilia 70910-900, Brazil; camilabiete@gmail.com; 2 Graduate Program in Public Health, Department of Nutrition, University of Brasilia, Brasilia 70910-900, Brazil; vivian.goncalves@unb.br; 3 Graduate Program in Public Health, Federal University of Minas Gerais, Belo Horizonte 31270-901, Brazil; arienecarmo@gmail.com

**Keywords:** pregnancy, food insecurity risk, depression, home-visiting program, parental practices

## Abstract

Food insecurity (FI) is a critical issue in developing countries, particularly in low-resource settings, where it can worsen women’s mental health. Psychosocial factors such as low household income, limited education, multiparity, and vulnerability are linked to depressive symptoms during pregnancy. Additionally, the family environment influences parental practices, which may impact mental health. This study evaluates the association of socioeconomic factors, parental practices, FI risk, and home visit frequency with depressive symptoms in pregnant women enrolled in the Happy Child Program (Programa Criança Feliz—PCF) in the Federal District, Brazil. In this cross-sectional study, 132 pregnant women monitored by PCF from May to July 2023 were assessed using a self-administered questionnaire for socioeconomic data, the two-item Triage for Food Insecurity (TRIA) instrument for FI risk, the Scale of Parental Beliefs and Early Childhood Care Practices, and the Beck Depression Inventory-II for depressive symptoms. Most participants were multiparous (87.9%), had low income (under 200 USD/month; 80.8%), presented depressive symptoms (67.4%) and were at risk of FI (81.8%). About half demonstrated adequate parental practices (50.8%) and received four home visits per month during pregnancy (54.5%). Women who received four PCF home visits had a lower prevalence of depressive symptoms compared to those with fewer visits (PR 0.76, 95% CI 0.59–0.98). No significant association was found between FI or parental practices and depressive symptoms. These findings suggest that the PCF home-visiting program may strengthen vulnerable families, support social networks, and improve mental health during pregnancy. Additionally, the results of this study highlight the need for targeted interventions aimed at reducing food insecurity and promoting mental health during pregnancy, particularly among socially vulnerable populations. Furthermore, they reinforce the importance of expanding access to home-visiting programs as an effective strategy to improve maternal mental health and well-being, while fostering healthier prenatal environments for both mothers and their children.

## 1. Introduction

Promoting the health and well-being of future generations should begin prior to conception and continue throughout pregnancy. Adverse outcomes in maternal health and fetal development, such as those caused by excessive stress and nutritional deficiencies, can have lasting effects that persist throughout the lifespan [1,2].

Women of reproductive age are particularly vulnerable to developing depression. Research has shown a strong association between stressful conditions, including food insecurity (FI), and the manifestation of depressive symptoms during pregnancy [3,4,5], as well as a reduced quality of life [6]. In the United States, a cross-sectional study of 1158 pregnant women from the National Health and Nutrition Examination Survey (NHANES) found that 19% of pregnant women with a family income ≤ 300% of the Federal Poverty Level (FPL) experienced FI [7]. In low-income countries such as Nigeria, a national survey of 3519 pregnant adolescents and adults revealed a 75% prevalence of FI [8]. In Brazil, the prevalence of FI among pregnant women has been estimated to range from 34.8% to 71.5% [9,10,11,12]. There is substantial evidence linking FI with depressive symptoms during pregnancy [13,14]. For instance, Fisher et al. (2012) reported a pooled prevalence of depression of 16% during pregnancy across nine countries, with nearly 20% in the first year postpartum in 17 low- and middle-income countries [15]. Additionally, evidence from Austin & Lumley (2003) suggests that depression during pregnancy is a predictor of postpartum depression [16].

FI during pregnancy has been consistently associated with mental health disorders such as depression [3,4]. Studies show that FI exacerbates these symptoms in pregnant women, particularly in socially vulnerable settings [5,13]. Laraia et al. (2022) reinforce this relation, demonstrating that exposure to FI is a significant linked to the development of depressive symptoms, suggesting that the lack of access to food can trigger or worsen these symptoms in low-income pregnant women [5]. These findings align with the results of Khoshgoo et al. (2020), who found that FI was associated with higher odds of depressive symptoms in Iranian pregnant women. The prevalence of elevated depressive symptoms was significantly higher among Iranian women experiencing FI and unemployment, while women with higher levels of education presented lower association rates [4]. Spariling et al. (2018) also showed that FI and poor household food consumption were associated with more than double the odds of depression in among women of reproductive age in rural Bangladesh [17].

The family environment significantly impacts maternal mental health [18,19]. Families experiencing poverty and economic hardship are more likely to experience elevated parental stress and inter-parental conflict, leading to inadequate parenting practices [20,21]. Such harsh parenting practices are linked to impaired mental health and a higher prevalence of depression and anxiety [22]. Therefore, understanding pregnant women’s knowledge about parental beliefs and practices is crucial for improving maternal and child health and well-being.

This context underscores the importance of government social policies and programs that protect vulnerable families. In 2016, Brazil launched the Happy Child Program (Programa Criança Feliz–PCF), a home-visiting initiative aiming to strengthen families and enhance intersectoral coordination to improve health and social services at the local, state, and national levels [23,24]. In 2019, the PCF was implemented in Brasília, Federal District, with home visits targeting families living in poverty, as identified in the national database of vulnerable populations [25]. The PCF aims to provide comprehensive social and health care to impoverished families, including those with pregnant women, children under the age of three, and children with disabilities under six years old. While some studies have evaluated the outcomes of families and children served by the PCF [26,27], few have focused on analyzing the factors associated with vulnerability and mental health among pregnant women in the Federal District, Brazil, who were enrolled in the program.

In this context, this study aims to investigate the association between the risk of FI, parental beliefs, the number of home visits, and depressive symptoms during pregnancy among women enrolled in the PCF.

## 2. Materials and Methods

### 2.1. Study Design and Study Settings

This observational cross-sectional study was conducted with women assisted during pregnancy by the PCF in the Federal District (FD), Brazil, from May to July 2023. The FD, located in the Center-West region of Brazil, includes Brasília, the nation’s capital. With a population of approximately 2,817,381 distributed across 35 administrative regions [28,29], the FD had 15.7% of its population living in poverty in 2021, defined as having an income below USD 5.50 per capita per day [30].

The PCF serves 3200 families across the 16 administrative regions of the Federal District, providing social and health care to families in poverty. The program schedules biweekly visits for pregnant women and weekly visits for families with children under six years old. It is important to note that approximately 55% of the pregnant women enrolled in the PCF already had other children receiving weekly visits, which explains why some individuals received more frequent visits. All pregnant women registered with the PCF between May and June 2023 were invited to participate in the study.

### 2.2. Sample

This study presents a census sample. All pregnant women enrolled in the PCF during pregnancy were contacted and invited to participate. The total list of pregnant women consisted of 231 women, of whom 132 agreed to participate, resulting in a participation rate of approximately 57%.

### 2.3. Eligibility Criteria

**Figure 1:**
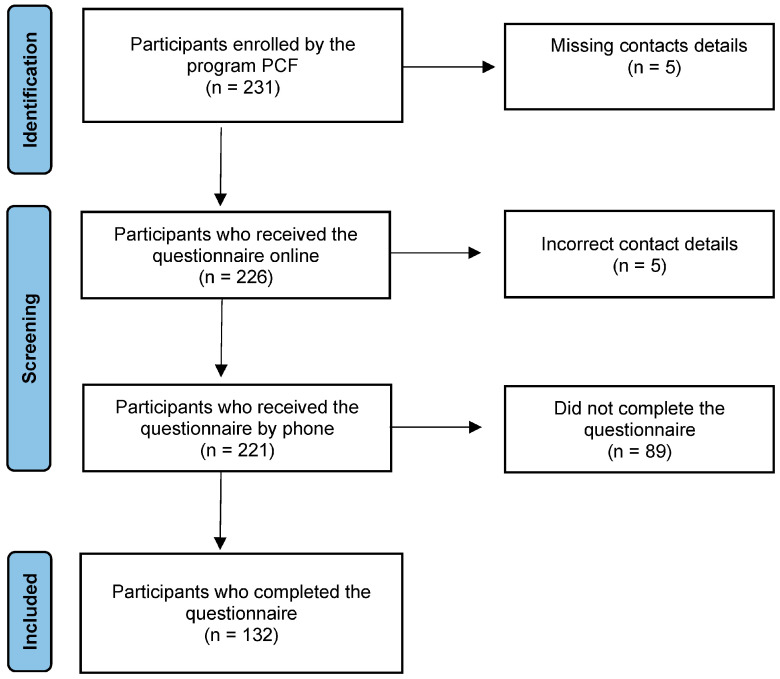
Flow diagram of individuals participating in the study.

Inclusion criteria for the study were being enrolled in the PCF during pregnancy and residing in one of the 16 administrative regions served by the program. The study included women who were currently pregnant as well as those who reported being in the postpartum period. Exclusion criteria comprised individuals who either did not respond to the questionnaire or declined to participate (see Figure 1).

### 2.4. Data Collection

The individuals who agreed to participate responded to an online questionnaire that included sociodemographic data (age, education level, ethnicity (color or race), marital status), economic data (monthly income, beneficiaries of social programs including or not cash transfer programs gestational data, health data during pregnancy (depression symptoms, illnesses during pregnancy), household food insecurity (HFI) and parenting skills data.

The assessment of the risk for FI was conducted using the two-item Triage for Food Insecurity (TRIA) instrument: (1) “In the last 3 months, did you run out of food before you had money to buy more food?” and (2) “In the last 3 months, you only ate some food that you had left, why did you run out of money?”. Individuals who responded positively to both questions were categorized as a risk for FI [31,32].

Depressive symptoms during pregnancy were analyzed using the Beck Depression Inventory-II, developed by Beck et al. (1996) [33], translated and validated in Brazil by Gomes-Oliveira et al. (2012) [34]. This questionnaire consists of 21 questions, with each item scored from 0 to 3 points. Individuals who obtained a score equal to or greater than 14 points were categorized as presenting depressive symptoms [35].

The assessment of parental skills was carried out using the Scale of Parental Beliefs and Early Childhood Care Practices (ECPPC) adapted to the gestational period [36]. The instrument evaluates parental practices, asking about primary care and stimulation. It consists of 18 items rated on a Likert scale in which participants assess the importance of care practices using the following options: (1) “not very important”, (2) “reasonably important”, (3) “somewhat important”, (4) “important”, and (5) “very important”. The result is obtained by adding the score, and the more important the practices and beliefs are considered, the higher the score assigned. The score can range from 18 to 90 points. Since there is no validated cut-off point to pregnant groups, the median value of the items was considered to categorize as adequate or non-adequate parental practices.

The PCF home visit frequency was categorized according to the number of monthly visits reported during pregnancy as four (4) or under four (>4).

### 2.5. Data Analysis

The descriptive analysis included the calculation of proportions and their respective 95% confidence intervals (95% CI) for the categorical variables of the study. The Pearson’s Chi-Square Test was performed to compare the proportions.

Crude and adjusted Poisson regression models with robust variance were carried out. Poisson regression with robust variance provides correct estimates and is a better alternative for analyzing cross-sectional studies with binary outcomes than logistic regression. The presence of depressive symptoms was the dependent variable, and PCF visit frequency (the number of PCF home visits), the risk of food insecurity, and the ECPPC adequation were the explanatory variables. The model was adjusted by the following variables: family income, previous pregnancy, gestational period, education, and self-reported skin color.

The Hosmer & Lemeshow test was used to check the fit of the final model. The prevalence ratio (PR) with a 95% confidence interval (95% CI) was used as the measure of effect. The data obtained were analyzed using Stata software version 17.0. The significance value of 5% (*p* < 0.05) was adopted for all analyses.

### 2.6. Ethical Aspects

To participate in the study, women accessed the Free and Informed Consent Form (TCLE) through an online form. By checking the “yes” option in the question “Do you agree with the terms above and accept to participate in the research?”, participants authorized the use of their data to carry out the research. The study received ethical approval by the Ethics Committee for Research with Human Beings of the Faculty of Health Sciences of the University of Brasília (CAAE: 32390620.0.0000.0030).

## 3. Results

The study included 132 perinatal women (Table 1) aged between 18 and 46 years. The majority identified as Black or Brown (76.34%; 95% CI, 68.23–82.89) and had completed high school. Among the participants, 50.83% (95% CI, 41.87–59.75) reported a monthly household income between USD 100.00 and USD 200.00, and a significant proportion were beneficiaries of the Family Grant Program (Programa Bolsa Família) (75%; 95% CI, 66.84–81.70). Of the women surveyed, 44.3% (95% CI, 35.9–52.9) were in the third trimester of pregnancy, 41.22% (95% CI, 33.05–49.91) were in the postpartum period, and 87% were multiparous. Of the sample, 74 women reported having other children enrolled in the PCF, with 54 receiving four visits per month and 20 receiving fewer than four.

**Figure 2:**
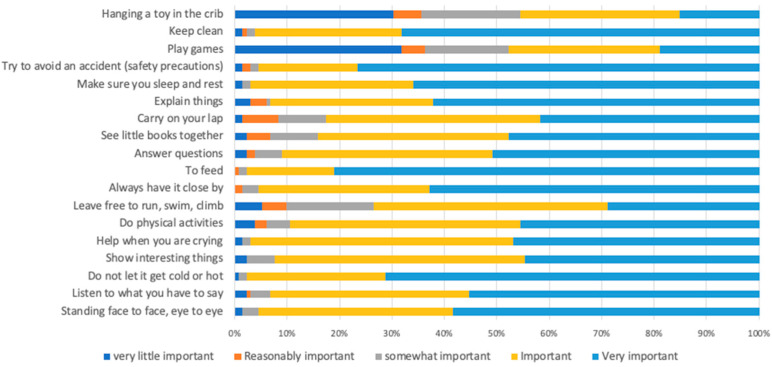
Percentage of parental practice responses according to the ECPPC.

Regarding exposure variables, most of the women were at risk of food insecurity (81.82%; 95% CI, 74.22–87.55), and half of them presented adequate parental practices (ECPPC > 79 points) (50.76%; 95% CI, 42.21–59.26) and received four PCF home visits monthly (54.55%; 95% CI, 45.92–62.90). The care practices highly considered as very important for individuals were “feeding” (81%) and “trying to avoid an accident (safety care)” (76.5%). Most care practices considered “not very important” were “playing games” (31.8%) and “hanging toys in the crib” (30.3%). The detailed percentage of parental practice responses is available in Figure 2. The depressive symptoms outcome variable presented a frequency of 67.42% (95% CI, 58.90–74.93).

A higher prevalence of Family Grant Program beneficiaries (91.7% versus 55.0%, *p* < 0.001) and multiparous women (94.4% versus 80.0%, *p* < 0.011) was observed among individuals who received four visits. A higher prevalence of low income was identified among those at risk of FI (52.5% versus 42.9%, *p* < 0.047) (Table 2).

According to the adjusted regression model presented in Table 3, the prevalence of depressive symptoms was 24% lower among those women who received four PCF home visits compared to those who received fewer visits (PR 0.76, 95% CI 0.59–0.98). No significant association between parental practice and risk for household food insecurity with depressive symptoms was found (*p* > 0.05).

## 4. Discussion

This cross-sectional study of vulnerable women found that 67.4% of those enrolled in the PCF during pregnancy exhibited symptoms of depression. This prevalence is notably higher than that reported in other studies of similar pregnant populations when stratified by factors such as skin color, education level, and household income. For instance, Suarte et al. (2021) reported a 52.5% prevalence of depression among pregnant women treated in a public hospital in the Federal District [37]. Another study conducted in the same region found a 45.2% prevalence of depression among pregnant women, though the sample had a higher educational level compared to our study population [38].

Other studies have reported perinatal depression rates of 21.9% [39] and 27.2% [40] among Brazilian pregnant women [41]. A recent systematic review highlighted that perinatal depression is more prevalent in lower-middle-income countries, with a pooled prevalence of 25.5% (95% CI, 23.8–27.1%; 197 studies from 23 countries, including 212,103 individuals), compared to high-income countries. This condition affects one in four perinatal women in low- and middle-income countries [42]. Our findings align with previous studies and emphasize the critical mental health challenges faced by vulnerable pregnant women enrolled in government social programs in Brazil. Although the exact causal factors for depression are not fully understood, identifying and addressing these factors is essential to reduce the burden of depression in vulnerable populations.

Lower education levels are associated with other socioeconomic disadvantages, such as low income and limited access to better-paying jobs, leading to a lower socioeconomic status [43]. Families living in poverty often have insufficient access to adequate food, both in quantity and quality, which contributes to the double burden of malnutrition [44]. This study observed a high prevalence of FI among low-income women. While FI is independently linked to mental health, Gundersen and Ziliak (2015) argue that FI is a more direct indicator of financial hardship, encompassing risk factors beyond low income, such as poor nutrition and social marginalization [45]. Although this study did not find a direct association between FI and worsening mental health during pregnancy, previous research has shown such a link [4,46].

A recent systematic review identified the PCF home-visiting program as an effective strategy for strengthening social support, developing parenting skills, and enhancing family bonds among vulnerable families in Brazil [27]. Another public home-visiting initiative, the Better Early Childhood Program (Programa Primeira Infância Melhor), has demonstrated improvements in responsive caregiving, particularly among low-income families [47]. Evaluations of large-scale parenting programs in Latin America have shown promising results in enhancing caregiving practices in countries such as Colombia [48], Mexico [49], and Peru [50], suggesting that home-visiting programs are able to improve low-income families’ quality of life.

Given the association between vulnerability and poor mental health outcomes, the impact of home-visiting programs on perinatal depression was explored in a scoping review by Tabb et al. (2023). The review, which included 5160 individuals, found that home-visiting programs can mitigate the effects of perinatal depression [51]. Social support is considered a key protective factor against maternal depression [52]. Our data indicate a 24% lower risk of depressive symptoms among women who received at least four home visits during pregnancy, suggesting that social support can help mitigate the negative impact of untreated maternal mental health issues during the perinatal period.

In Brazil, the Family Grant Program (Programa Bolsa Família—PBF), a cash transfer initiative, has contributed to reducing financial vulnerability among pregnant women in low-income situations, regardless of family structure [53]. The PBF plays a crucial role in breaking the cycle of poverty and provides a protective effect on maternal and child health [54]. Although the PCF is a social program that does not provide direct income support, it was observed that participants who received more home visits were also PBF beneficiaries (91.7% vs. 55.0%, *p* < 0.001) and were more likely to be multiparous (94.4% vs. 80.0%, *p* < 0.011). While PBF eligibility was not a criterion for PCF enrollment, the overlap between the target populations of these two programs meant that a significant proportion of women were beneficiaries of both.

Research with middle-class Brazilian mothers has shown an association between negative parenting, current maternal depression, and behavioral problems in children [55]. Faisal-Cury et al. (2009) found that 69% of low-income pregnant women experienced co-occurring depressive symptoms and anxiety [56]. Depressive symptoms can reduce a mother’s responsiveness, leading to less engaged mother–child interactions and an increased use of inadequate parenting practices [57]. One goal of home-visiting programs is to enhance parental skills, boost confidence, and create more stimulating home environments while reducing the use of physical punishment and yelling [58], even in challenging circumstances such as poverty and vulnerability [59]. Therefore, we believe that positive parenting can serve as a protective factor against the negative impacts of maternal depression. An analysis of the Better Early Childhood Program, implemented in Southern Brazil for nearly two decades, showed that parental effectiveness was most pronounced when the program began during pregnancy and targeted poorer families [60].

Although few studies have reported reductions in perinatal depression among low-income pregnant women enrolled in home-visiting programs [61], our study found that women who received four or more visits from the PCF had a lower prevalence of depressive symptoms. Additionally, there was a significant association between the number of visits, receipt of the Family Grant Program benefits, and multiparity. However, no association was observed between the risk of FI and depressive symptoms. The study by Ammerman et al. (2009) showed that low-income pregnant women in the United States enrolled in the Every Child Succeeds (ECS) home-visiting program from the 20th week of gestation experienced a significant reduction in depressive symptoms after being followed by the program. This longitudinal study revealed that 45.3% of pregnant women exhibited significant clinically depressive symptoms at some time during the follow-up period. The study showed that monitored women received an average of 22.67 home visits over the 9 months of follow-up [62]. Additionally, a scoping review conducted by Tabb et al. (2022) investigated the impact of home visits on perinatal depression and found that home-visiting programs can reduce the effects of depression. The evaluated articles reported a varied frequency of home visits. The review also revealed that home visits starting in the prenatal period are more effective in preventing depression compared to those starting postpartum. However, it is important to note that the home-visiting programs evaluated in the scoping review provide medical treatment to participants, which may influence the final results after the follow-up period [51].

Given the challenges associated with large-scale programs like the PCF, establishing strong bonds between home visitors and vulnerable families, particularly pregnant women, is crucial for addressing family needs. Increasing the number of home visits to pregnant women may be an effective strategy for improving maternal mental health and promoting better parenting practices.

This study is the first to document depressive symptoms during pregnancy among vulnerable women enrolled in a Brazilian PCF social program. The use of rigorous methodological tools strengthens our findings. Since the sample includes all eligible participants during the gestational period, and a comprehensive view of the program’s target population is provided. This approach ensures that the results reflect the experiences of the pregnant women enrolled, thereby strengthening the representativeness of the available data. However, the study has several limitations: (i) the cross-sectional design limits the ability to establish causal relationships; (ii) the sample reflects results from the PCF home-visiting program in Brazil, which may limit the generalizability of our findings; (iii) the study did not account for whether participants received treatment for depression during pregnancy, which may have influenced the association between FI and depressive symptoms; and (iv) the use of self-administered questionnaires may introduce reliability bias, as responses depend on the participants’ accuracy.

## 5. Conclusions

The majority of women enrolled in the PCF during pregnancy exhibited a higher risk of depressive symptoms and food insecurity (FI) yet demonstrated adequate parenting practices. Notably, receiving at least four PCF home visits was associated with a 24% lower prevalence of depressive symptoms during pregnancy. Further research is needed to explore the potential link between food insecurity and depressive symptoms in vulnerable groups. These findings underscore the importance of promoting and reinforcing social policies that ensure the health and well-being of women during pregnancy. Furthermore, the results highlight the importance of expanding access to home-visiting programs as an effective strategy to promoting maternal mental health and well-being. Future research to explore the effectiveness of such programs in different regional and social contexts is recommended. Additionally, longitudinal studies to assess the long-term effects of home visits on the mental health of pregnant women in socially vulnerable situations need to be conducted.

## Figures and Tables

**Table 1:** Characteristics of women enrolled in the PCF during pregnancy. Federal District, Brazil, 2023 (n = 132).

Variables	n	%	95% CI
**Age**			
≤19 years	9	6.9	3.6; 12.8
20 to 24 years	34	26.2	19.3; 34.5
25 to 29 years	27	20.8	14.6; 28.7
30 to 34 years	31	23.8	17.3; 32.0
≥35 years	29	22.3	15.9; 30.3
**Gestational Period**			
1st Trimester	1	0.8	0.1; 5.3
2nd Trimester	18	13.7	8.8; 20.8
3rd Trimester	58	44.3	35.9; 52.9
Postpartum	54	41.2	33.1; 49.9
**Cash Transfer Program-enrolled**			
No	33	25.0	18.3; 33.2
Yes	99	75.0	66.8; 81.7
**Educational Level**			
Elementary School	33	25.2	18.4; 33.4
High school	89	67.9	59.4; 75.4
University education	9	6.9	3.6; 12.8
**Ethnicity (color or race)**			
White	24	18.3	12.6; 25.9
Black/Brown (Multiracial)	100	76.3	68.2; 82.9
Yellow (Asian)	4	3.1	1.1; 7.9
Indigenous	3	2.3	0.7; 6.9
**Monthly Household Income (USD)**			
Up to 100.00	36	30.0	22.4; 38.9
From 100.00 to 200.00	61	50.8	41.9; 59.8
Over 200.00	23	19.2	13.0; 27.3
**Previous Pregnancy**			
Primiparous	16	12.1	7.5; 18.9
Multiparous	116	87.9	81.1; 92.5
**Symptoms of Depression**			
No	43	32.6	25.1; 41.1
Yes	89	67.4	58.9; 74.9
**Parental Practice**			
Non-adequate	65	49.2	40.7; 57.8
Adequate	67	50.8	42.2; 59.3
**Food Insecurity Risk**			
No	24	18.2	12.5; 25.8
Yes	108	81.8	74.2; 87.6
**PCF Visit Frequency**			
<4	60	45.5	37.1; 54.1
4	72	54.5	45.9; 62.9

**Table 2:** Explanatory variables and depressive symptoms, food insecurity risk, parental practices, and visit frequency of women enrolled in the PCF during pregnancy. Federal District, Brazil, 2023 (n = 132).

Explanatory Variables	No Depression Symptoms		CI 95%	Depressive Symptoms		CI 95%	*p*-Value	No FI Risk		CI 95%	FI Risk		CI 95%	*p*-Value
	n	%		n	%			n	%		n	%		
**Age**							0.549							0.145
≤19 years	3	7.1	(2.2–20.5)	6	6.8	(3.1–14.5)		1	4.5	(0.6–28.6)	8	7.4	(3.7–14.2)	
20 to 24 years	12	28.6	(16.7–44.4)	22	25.0	(17.0–35.2)		10	45.5	(25.5–67.0)	24	22.2	(15.3–31.1)	
25 to 29 years	5	11.9	(4.9–26.1)	22	25.0	(17.0–35.2)		3	13.6	(4.2–36.5)	24	22.2	(15.3–31.1)	
30 to 34 years	11	26.2	(14.9–41.9)	20	22.7	(15.1–32.8)		6	27.3	(12.2–50.4)	25	23.1	(16.1–32.1)	
≥35 years	11	26.2	(14.9–41.9)	18	20.5	(13.2–30.3)		2	9.1	(2.1–31.9)	27	25.0	(17.7–34.1)	
**Gestational** **Period**							0.065							
1st Trimester	1	2.3	(0.3–15.5)	0	0.0	0.00		0	0.0	0.00	1	0.9	(0.1–6.5)	0.523
2nd Trimester	10	23.3	(12.8–38.6)	8	9.1	(4.6–17.3)		5	20.8	(8.5–42.7)	13	12.1	(7.1–19.9)	
3rd Trimester	16	37.2	(23.9–52.8)	42	47.7	(37.4–58.3)		8	33.3	(17.0–55.0)	50	46.7	(37.4–56.3)	
Postpartum	16	37.2	(23.9–52.8)	38	43.2	(33.1–53.8)		11	45.8	(26.6–66.4)	43	40.2	(31.3–49.8)	
**Cash** **Transfer Program-enrolled**							0.335							0.297
No	13	30.2	(18.2–45.9)	20	22.5	(14.9–32.4)		8	33.3	(17.0–55.1)	25	23.1	(16.1–32.1)	
Yes	30	69.8	(54.1–81.9)	69	77.5	(67.6–85.1)		16	66.7	(45.0–83.1)	83	76.9	(67.9–83.9)	
**Schooling**							0.938							0.561
Elementary School	10	23.3	(12.8–38.6)	23	26.1	(17.9–36.4)		4	16.7	(6.1–38.3)	29	27.1	(19.5–36.4)	
High school	30	69.8	(54.1–81.9)	59	67.0	(56.5–76.2)		18	75.0	(53.1–88.8)	71	66.4	(56.8–74.7)	
University education	3	7.0	(2.2–20.1)	6	6.8	(3.1–14.5)		2	8.3	(1.9–29.5)	7	6.5	(3.1–13.2)	
**Ethnicity** **(color or race)**							0.181							
White	6	14.0	(6.3–28.3)	18	20.5	(13.2–30.3)		5	20.8	(8.5–42.7)	19	17.8	(11.6–26.3)	
Black/Brown (Multiracial)	37	86.0	(71.8–93.8)	63	71.6	(61.2–80.1)		18	75.0	(53.1–88.8)	82	76.6	(67.6–83.8)	
Yellow (Asian)	0	0.0	0.00	4	4.5	(1.7–11.6)		0	0.0	0.00	4	3.7	(1.4–9.6)	
Indigenous	0	0.0	0.00	3	3.4	(1.1–10.2)		1	4.2	(0.5–26.5)	2	1.9	(0.5–7.3)	
**Monthly Household** **Income (USD)**							0.406							0.047
Up to 100.00	14	36.8	(22.8–53.6)	22	26.8	(18.3–37.6)		4	19.0	(6.9–42.9)	32	32.3	(23.8–42.3)	
From 100.00 to 200.00	16	42.1	(27.2–58.6)	45	54.9	(43.9–65.4)		9	42.9	(23.0–65.3)	52	52.5	(42.6–62.3)	
Over 200.00	8	21.1	(10.6–37.4)	15	18.3	(11.3–28.3)		8	38.1	(19.4–61.1)	15	15.2	(9.3–23.8)	
**Previous** **Pregnancy**														0.148
Primiparous	8	18.6	(9.4–33.5)	8	9.0	(4.5–17.1)	0.113	5	20.8	(8.5–42.7)	11	10.2	(5.7–17.6)	
Multiparous	35	81.4	(66.5–90.6)	81	91.0	(82.9–95.5)		19	79.2	(57.3–91.5)	97	89.8	(82.4–94.3)	
**Explanatory Variables**	**Non-Adequate** **Parental Practices**		**CI 95%**	**Adequate Parental** **Practices**		**CI 95%**	***p*** **-Value**	**PCF Visit** **Frequency (<4)**		**CI 95%**	**PCF Visit** **Frequency (4)**		**CI 95%**	***p*** **-Value**
	**n**	**%**		**n**	**%**			**n**	**%**		**n**	**%**		
**Age**														0.127
≤19 years	3	4.7	(1.5–13.8)	6	9.1	(4.1–19.0)	0.653	4	6.9	(2.6–17.3)	5	6.9	(2.9–15.8)	
20 to 24 years	17	26.6	(17.0–38.9)	17	25.8	(16.5–37.8)		17	29.3	(18.9–42.5)	17	23.6	(15.1–35.0)	
25 to 29 years	15	23.4	(14.5–35.6)	12	18.2	(10.5–29.6)		16	27.6	(17.5–40.7)	11	15.3	(8.6–25.7)	
30 to 34 years	17	26.6	(17.0–38.9)	14	21.2	(12.9–32.9)		8	13.8	(7.0–25.5)	23	31.9	(22.1–43.7)	
≥35 years	12	18.8	(10.9–30.4)	17	25.8	(16.5–37.8)		13	22.4	(13.3–35.2)	16	22.2	(14.0–33.5)	
**Gestational** **Period**							0.394							0.418
1st Trimester	0	0.0	0.00	1	1.5	(0.2–10.3)		0	0.0	0.00	1	1.4	(0.2–9.6)	
2nd Trimester	10	15.4	(8.4–26.5)	8	12.1	(6.1–22.7)		6	10.0	(4.5–20.8)	12	16.9	(9.8–27.7)	
3rd Trimester	25	38.5	(27.3–51.0)	33	50.0	(38.0–62.0)		30	50.0	(37.3–62.6)	28	39.4	(28.6–51.4)	
Postpartum	30	46.2	(34.3–58.5)	24	36.4	(25.5–48.8)		24	40.0	(28.2–53.0)	30	42.3	(31.2–54.2)	
**Cash Transfer Program-enrolled**							0.615							0.001
No	15	23.1	(14.3–35.1)	18	26.9	(17.5–38.9)		27	45.0	(32.7–57.9)	6	8.3	(3.7–17.5)	
Yes	50	76.9	(64.9–85.7)	49	73.1	(61.1–82.5)		33	55.0	(42.1–67.3)	66	91.7	(82.5–96.3)	
**Schooling**							0.275							0.416
Elementary School	13	20.0	(0.1–31.7)	20	30.3	(20.3–42.6)		14	23.3	(14.2–35.9)	19	26.8	(17.6–38.4)	
High school	46	70.8	(58.4–80.7)	43	65.2	(52.7–75.8)		40	66.7	(53.6–77.6)	49	69.0	(57.2–78.8)	
University education	6	9.2	(4.1–19.3)	3	4.5	(1.4–13.4)		6	10.0	(4.5–20.8)	3	4.2	(1.3–12.5)	
**Ethnicity (color or race)**							0.281							0.463
White	15	23.1	(14.3–35.1)	9	13.6	(7.2–24.4)		9	15.0	(7.9–26.7)	15	21.1	(13.0–32.4)	
Black/Brown (Multiracial)	45	69.2	(56.8–79.4)	55	83.3	(72.1–90.6)		46	76.7	(64.1–85.8)	54	76.1	(64.6–84.7)	
Yellow (Asian)	3	4.6	(1.5–13.6)	1	1.5	(0.2–10.3)		3	5.0	(1.6–14.7)	1	1.4	(0.2–9.6)	
Indigenous	2	3.1	(0.8–11.8)	1	1.5	(0.2–10.3)		2	3.3	(0.8–12.7)	1	1.4	(0.2–9.6)	
**Monthly Household Income (USD)**							0.231							0.903
Up to 100.00	18	30.0	(19.6–43.0)	18	30.0	(19.6–43.0)		16	28.1	(17.8–41.3)	20	31.7	(21.3–44.4)	
From 100.00 to 200.00	27	45.0	(32.7–57.9)	34	56.7	(43.7–68.8)		30	52.6	(39.5–65.4)	31	49.2	(36.9–61.6)	
Over 200.00	15	25.0	(15.5–37.7)	8	13.3	(6.7–24.8)		11	19.3	(10.9–31.9)	12	19.0	(11.0–30.9)	
**Previous** **Pregnancy**							0.948							0.011
Primiparous	8	12.3	(6.2–23.0)	8	11.9	(6.0–22.3)		12	20.0	(11.6–32.3)	4	5.6	(2.1–14.1)	
Multiparous	57	87.7	(77.0–93.8)	59	88.1	(77.7–94.0)		48	80.0	(67.7–88.4)	68	94.4	(85.9–97.9)	

**Table 3:** Association between parenting, number of visits PCF, and risk for FI and depressive symptoms (n = 132).

Variables	PR Crude (95% CI)	*p* Value *	PR Adjusted (95% CI) **	*p* Value *
**Parental Practices**				
Non-adequate parental practices	(ref)			
Adequate parental practices	1.05 (0.83–1.34)	0.664	1.07 (0.85–1.35)	0.548
**Food Insecurity Risk**				
No	(ref)			
Yes	1.30 (0.88–1.92)	0.188	1.27 (0.85–1.89)	0.237
**PCF Visit Frequency**				
<4	(ref)			
4	0.81 (0.64–1.03)	0.089	0.76 (0.59–0.98)	0.032

* Poisson Regression with robust variance. ** Model adjusted by the following variables: household income, previous pregnancy, gestational period, schooling, and self-reported skin color. Notes: Confidential Interval (CI), Prevalence Ratio (PR), Parental Beliefs and Early Childhood Care Practices (ECPPC).

## Data Availability

Data is contained within the article.
